# A Tracer Bolus Method for Investigating Glutamine Kinetics in Humans

**DOI:** 10.1371/journal.pone.0096601

**Published:** 2014-05-08

**Authors:** Maiko Mori, Marie Smedberg, Maria Klaude, Inga Tjäder, Åke Norberg, Olav Rooyackers, Jan Wernerman

**Affiliations:** Department of Anesthesiology and Intensive Care Medicine, Karolinska University Hospital Huddinge, Karolinska Institutet, Stockholm, Sweden; National Institute of Agronomic Research, France

## Abstract

Glutamine transport between tissues is important for the outcome of critically ill patients. Investigation of glutamine kinetics is, therefore, necessary to understand glutamine metabolism in these patients in order to improve future intervention studies. Endogenous glutamine production can be measured by continuous infusion of a glutamine tracer, which necessitates a minimum measurement time period. In order to reduce this problem, we used and validated a tracer bolus injection method. Furthermore, this method was used to measure the glutamine production in healthy volunteers in the post-absorptive state, with extra alanine and with glutamine supplementation and parenteral nutrition. Healthy volunteers received a bolus injection of [1-^13^C] glutamine, and blood was collected from the radial artery to measure tracer enrichment over 90 minutes. Endogenous rate of appearance (endoR_a_) of glutamine was calculated from the enrichment decay curve and corrected for the extra glutamine supplementation. The glutamine endoR_a_ of healthy volunteers was 6.1±0.9 µmol/kg/min in the post-absorptive state, 6.9±1.0 µmol/kg/min with extra alanyl-glutamine (p = 0.29 versus control), 6.1±0.4 µmol/kg/min with extra alanine only (p = 0.32 versus control), and 7.5±0.9 µmol/kg/min with extra alanyl-glutamine and parenteral nutrition (p = 0.049 versus control). In conclusion, a tracer bolus injection method to measure glutamine endoR_a_ showed good reproducibility and small variation at baseline as well as during parenteral nutrition. Additionally, we showed that parenteral nutrition including alanyl-glutamine increased glutamine endoR_a_ in healthy volunteers, which was not attributable to the alanine part of the dipeptide.

## Introduction

Glutamine is the most abundant amino acid in the body, in both plasma and the free intracellular amino acid pool in skeletal muscle. Skeletal muscle is regarded to be the main producer of glutamine, and contains 90% of the whole body free glutamine pool [1]. Glutamine is also one of the most important amino acids during metabolic stress, and is considered a conditionally essential amino acid during critical illness. Glutamine is utilized as an energy substrate and a precursor for nucleotides [2], especially in rapidly dividing intestinal epithelial cells [3] and immune cells [4], which are important during septic stress. It can also be a precursor for the biosynthesis of glutathione and a possible inducer of heat shock protein [5], which are important for protection from infection or oxidative stress.

Low plasma glutamine level at admission to the ICU is an independent predictor for mortality, suggesting that a decreased availability of glutamine may lead to a less favorable outcome [6], [7]. Consumption of glutamine exceeding the endogenous production during critical illness, may be the background behind the depletion of glutamine levels in plasma observed in ICU patients. Exogenous glutamine supplementation reduces mortality, infectious morbidity and length of ICU stay [8], but recently a study showed of harm following administration of large doses [9]. We therefore need to understand the relation between glutamine plasma concentration, endogenous glutamine production and glutamine availability during critical illness better to be able to design intervention studies in the future.

As highlighted previously, glutamine transport between tissues is important in critically ill patients, and therefore measurement of glutamine kinetics will enable us to better understand glutamine metabolism. In vivo endogenous glutamine production can be measured by tracer dilution methods. Glutamine kinetics has been studied previously by continuous infusion of labeled glutamine [10]. The large intracellular free glutamine pool has a limitetd exchangablility, but still a steady state may be achieved in the immediate exchangeable pool which may give reproducible kinetic measurements [11].

The purpose of the present study is to introduce an alternative technique, a bolus injection method, followed by obtaining a tracer enrichment decay curve, in order to facilitate repeated measurements during shorter time periods. By sampling blood frequently, we analyzed the tracer enrichment in plasma over time, and calculated glutamine endogenous production using a single pool model. Our purpose was to establish this bolus method as a single-pool estimate for screening purposes. Initially we chose to estimate glutamine endogenous production in healthy subjects. In addition, the effects of alanyl-glutamine with and without parenteral nutrition supplementation, and finally of alanine alone on endogenous glutamine production were examined.

## Methods

### Materials

[1-^13^C] glutamine and [^13^C5] glutamine were purchased from Cambridge Isotope Laboratories (Andover, MA, USA), N-methyl-N-(tertiary-butyldimethylsilyl) trifluoroacetamide was obtained from Regis Technologies (Morton Grove, IL, USA) and pure alanine was purchased from Ajinomoto (Chuo, Tokyo, Japan). Extra glutamine was given as a alanyl-glutamine (ala-gln) dipeptide, Dipeptiven, Fresenius Kabi (Uppsala, Sweden).

### Subjects

Healthy volunteers were recruited according to general physical examination and standard blood analyses. 32 healthy volunteers (18 males and 14 females) were included in the studies. Eight subjects participated in the two pilot studies, 17 participated in the ala-gln with and without nutrition study and 7 in the alanine study. All the study protocols conformed to the ethical guidelines of the 1975 Declaration of Helsinki and were approved by the Regional Ethical Review Board, Stockholm, Sweden. All healthy volunteers gave informed consent in writing to participate in the studies after receiving both oral and standardized written information approved by the Ethical Committee of Karolinska Instituet, Stockholm, Sweden.

### Study protocol

Catheters were inserted in two forearm veins; one for glutamine tracer bolus injection, and one for alanyl-glutamine, parenteral nutrition and/or alanine infusion. An additional catheter was inserted in the radial artery for blood sampling.

### Dose finding study

The purpose of this first pilot study was to find a dose for the glutamine tracer that allowed accurate tracer determination in plasma and no or minimal effects on glutamine concentrations. Four male healthy subjects were included in the study; age 26–35 years, body weight 80–100 kg. The subject received a bolus injection of [1-^13^C] glutamine at 1.5, 3 or 6 mg/kg, and blood was collected as described below. Samples were analyzed for glutamine concentrations and glutamine tracer enrichments. For the highest dose, also insulin levels were measured.

### Variation study

The purpose of the second pilot study was to determine the coefficient of variation for the analyses and the intra-individual variation for glutamine kinetics. Two studies per subject were performed in 4 female healthy subjects; age 22–49 years, body weight 55–80 kg, with 3 to 12 days in between to determine the variation and reproducibility within the subjects. All subjects received a bolus injection of [1-^13^C] glutamine (3 mg/kg, and blood was collected as described below. The samples of subjects 3 and 4 were analyzed for glutamine tracer enrichment in duplicate to also determine the assay coefficient of variation.

### Effect of alanyl-glutamine supplementation and parenteral feeding

The purpose of the study was to establish the endogenous rate of appearance (endoR_a_) of glutamine in control subjects using our new approach and to determine the effects of glutamine supplementation with and without parenteral nutrition on the endoR_a_ of glutamine. Seventeen healthy subjects were included in this study, 5 subjects (male/female 3/2, age 26–41 years, body weight 53–88 kg) for the control group (CON), 5 subjects (male/female 2/3, age 20–53 years, body weight 72–98 kg) received alanyl-glutamine supplementation without parenteral nutrition (GLN) and 6 subjects (male/female 4/2, age 21–40 years, body weight 65–110 kg) received alanyl-glutamine supplementation with parenteral nutrition (GLN+TPN). One subject of the GLN group was excluded due to violation of the study protocol. All subjects received a bolus injection of [1-^13^C]-glutamine (3 mg/kg) and thereafter blood was collected for 90 minutes. The ala-gln supplementation group received a continuous 25 mg/kg/h Dipeptiven infusion from 3 hours before bolus injection of [1-^13^C] glutamine until the end of the study. Parenteral nutrition Kabiven (Fresenius Kabi; 1.3 kcal/kg/h) (GLN+TPN) or saline (GLN) was continuously infused during the same time. On the data collected for the CON group, simulations over several sampling protocols were done.

### Alanine study

The purpose of this additional study was to determine whether the alanine given as part of the alanyl-glutamine supplementation had any effect on glutamine endoR_a_. Out of 8 recruited subjects one was excluded at an early stage since we were unable to insert an arterial catheter. The 7 subjects (male/female 5/2, age 24–44 years, body weight 50–92 kg) received a bolus injection of [1-^13^C]-glutamine (3 mg/kg) and thereafter blood was collected for 90 minutes. Next, a 4-hour continuous infusion of alanine at 9 mg/kg/h was started. The subjects received another bolus injection of [1-^13^C]-glutamine (3 mg/kg) 2.5 hours into the alanine infusion and blood was collected again for 90 minutes.

### Blood collection

Blood was collected from the radial artery. Two baseline samples were collected before the bolus injection of the tracer. After the bolus injection of [1-^13^C] glutamine (3 mg/kg), blood was collected every 30 seconds up to 10 min, every 1 min up to 30 min, every 3 min up to 90 min.

### Amino acid analysis

Glutamine tracer enrichment was analyzed by a gas chromatography-mass spectrometry (GC-MS) method described previously [12]. To suppress plasma glutamine converting to glutamate, ammonium formate was added to the plasma, which was then deproteinised by methanol. After centrifugation, the supernatant was dried in a rotary evaporator, derivatized by N-methyl-N-(tertiary-butyldimethylsilyl) trifluoroacetamide (MTBSTFA), and run by GC-MS. Mass to charge ratio (m/z) of 431 and 432 were measured for glutamine and [1-^13^C] glutamine respectively.

Glutamine concentration was analyzed by GC-MS by adding [^13^C5] glutamine as an internal standard and analyzing m/z 436.

### Calculations

Whole body rate of appearance (R_a_) of glutamine (µmol/kg/min) was calculated by a single pool model:



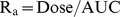



In which Dose is the amount of the tracer injected and AUC is the area under the curve of the atom percent excess (APE) versus time. The infusion of glutamine from the alanyl-glutamine was subtracted from the R_a_ to obtain the endoR_a_ of glutamine.

### Statistical analysis

All analyses were performed using GraphPad Prism software (version 5, GraphPad Software, Inc. La Jolla, CA). Variation study was analyzed by paired-T test. The effect of alanyl-glutamine supplementation and parenteral feeding was analyzed using ANOVA followed by Dunnett post-hoc tests. The effect of alanine alone was evaluated by Student's t-test for paired samples. A probability value of p<0.05 was considered statistically significant.

## Results

### Dose finding study

**Figure 1:**
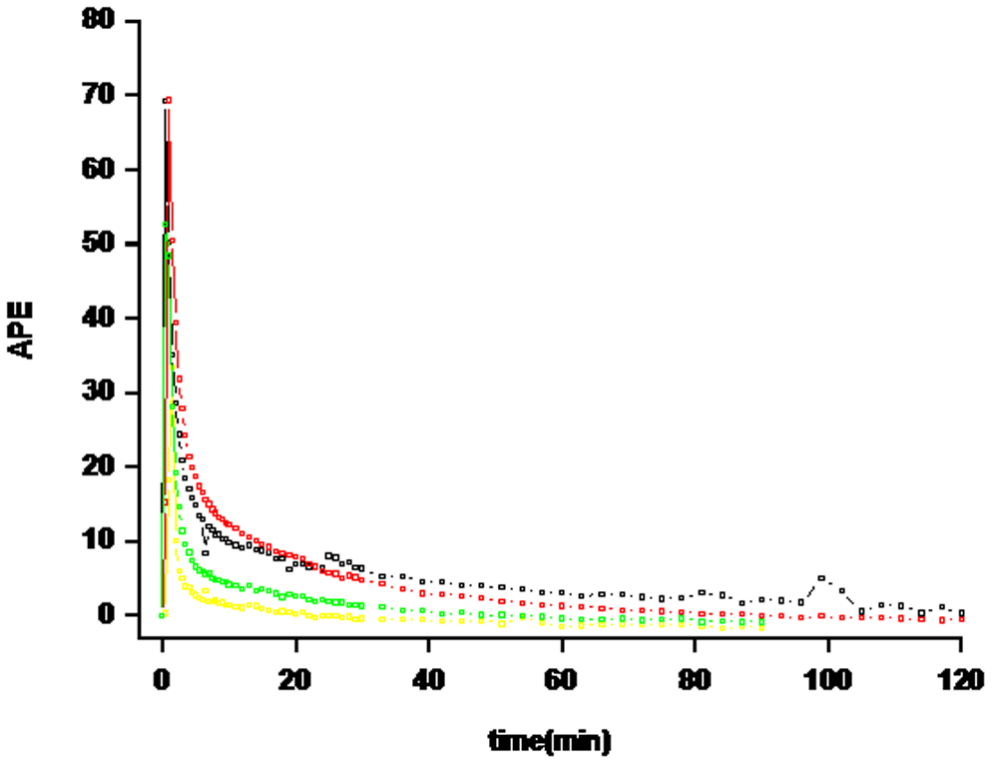
Glutamine APE (atom percent excess) over 90–120 min after bolus injection of 1.5 (blue symbols), 3 (green symbols), or 6 (black and red symbols) mg/kg of [1-^13^C] glutamine in healthy subjects (n = 4).

**Figure 2:**
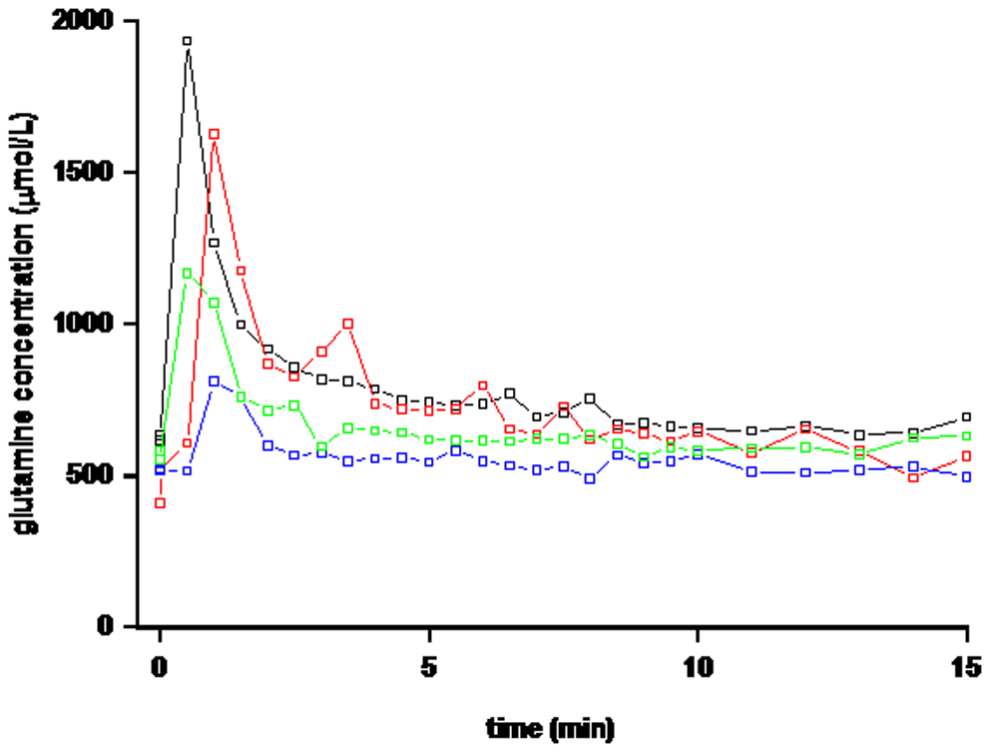
Plasma glutamine concentration over the initial 15(blue symbols), 3 (green symbols), or 6 (black and red symbols) mg/kg of [1-^13^C] glutamine in healthy subjects (n = 4). The decay-curve for 3 mg/kg reaches undetectable levels already after 60 minutes.

To determine the dose of [1-^13^C] glutamine, subjects received a bolus injection of 1.5, 3 or 6 mg/kg [1-^13^C] glutamine. This bolus injection of glutamine tracer resulted in an initial increase in the tracer enrichment which declined over time in an exponential curve (Figure 1). Glutamine concentration increased with all three doses in a dose dependent fashion (Figure 2). Concentrations were double after the 3 mg/kg and triple after the 6 mg/kg bolus. All concentrations were back at baseline within 5 minutes. Insulin concentration measured in the samples with the 6 mg/kg dose did not increase from baseline at any time point (data not shown). From these pilot data we decided to use a dose of 3 mg/kg in further studies since this gave us enrichments that were detectable over 60 minutes, and only increased glutamine concentration slightly during the first 5 minutes.

### Variation study

**Figure 3:**
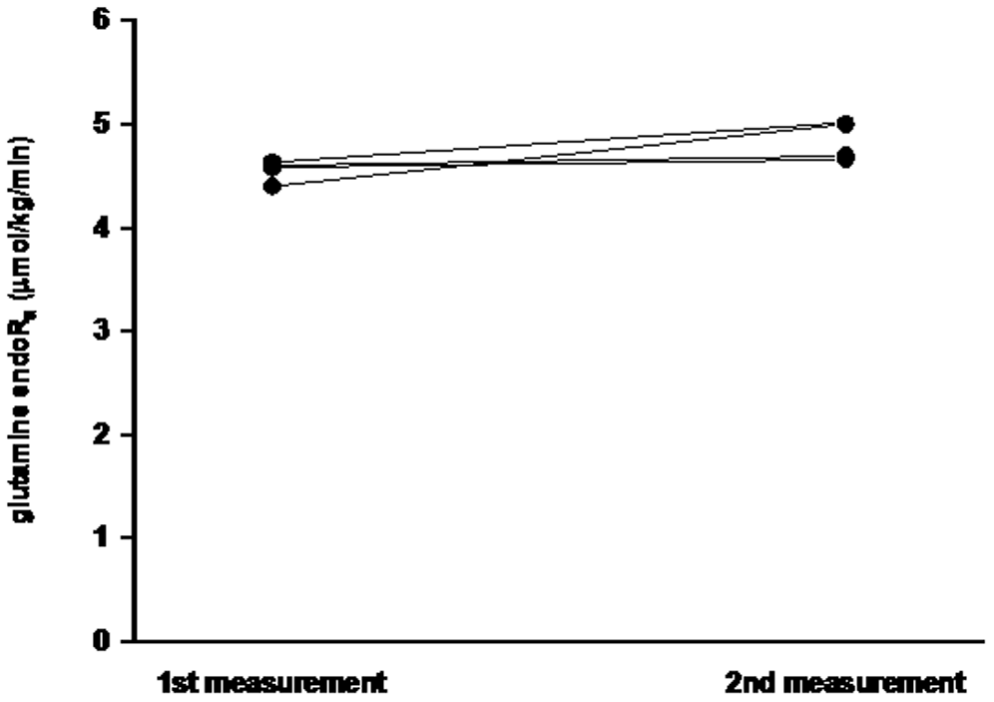
Endogenous glutamine R_a_ in healthy subjects (n = 4) studied repeatedly on 2 occasions in the postabsorptive state 3–12 days apart.

Glutamine endoR_a_ was calculated by a single pool model. All four subjects had glutamine endoR_a_ in the range of 4.4 to 5.1 µmol/kg/min (Figure 3 and Table S1). Glutamine endoR_a_ was similar in the 2 measurements performed on each subject, with no significant difference (P = 0.195). The CV between 2 studies was 5.5%. In 4 studies of 2 subjects, enrichment was analyzed twice by GC-MS for each study, and the analytical CV was 4.1%.

### Sampling protocol

On data from the subjects in the CON group below (n = 5), simulations over alternative sampling protocols were performed (Figure S1). For best accuracy both peak and tail needed to be well characterized. Both less frequent sampling during peak and less frequent sampling and/or shorter sampling period during tail resulted in differences from default sampling protocol that were statistically significant.

### Effect of alanyl-glutamine supplementation and parenteral feeding

**Figure 4:**
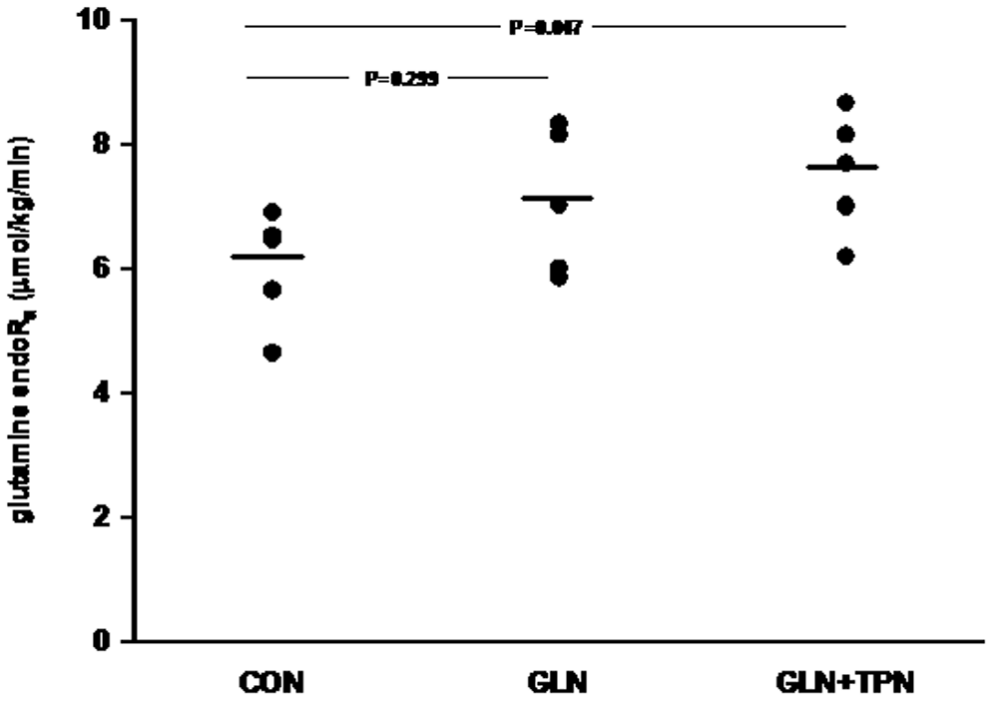
Endogenous glutamine R_a_ in healthy subjects given alanyl-glutamine supplementation and parenteral feeding. CON (n = 5) given no alanyl-glutamine or parenteral feeding, GLN (n = 5) given intravenous alanyl-glutamine only, and GLN+TPN (n = 6) given intravenous alanyl-glutamine and parenteral feeding.

Glutamine levels in the 2 groups receiving the extra glutamine supplementation were significantly higher than in the control group (CON; 517±71 µM, GLN; 775±49 µM, GLN+TPN; 740±92 µM, GLN versus CON P = 0.0001, GLN+TPN versus CON P = 0.0003). Glutamine endoR_a_ in the control group which received neither Ala-gln supplementation nor parenteral feeding was 6.1±0.9 µmol/kg/min (Figure 4 and Table S1). With alanyl-glutamine supplementation and extra parenteral feeding, glutamine endoR_a_ was higher (P = 0.047 versus CON), with 7.5±0.9 µmol/kg/min, while only alanyl-glutamine supplementation had no effect on glutamine endoR_a_ (6.9±1.0 µmol/kg/min, P = 0.299 versus CON).

### Alanine study

**Figure 5:**
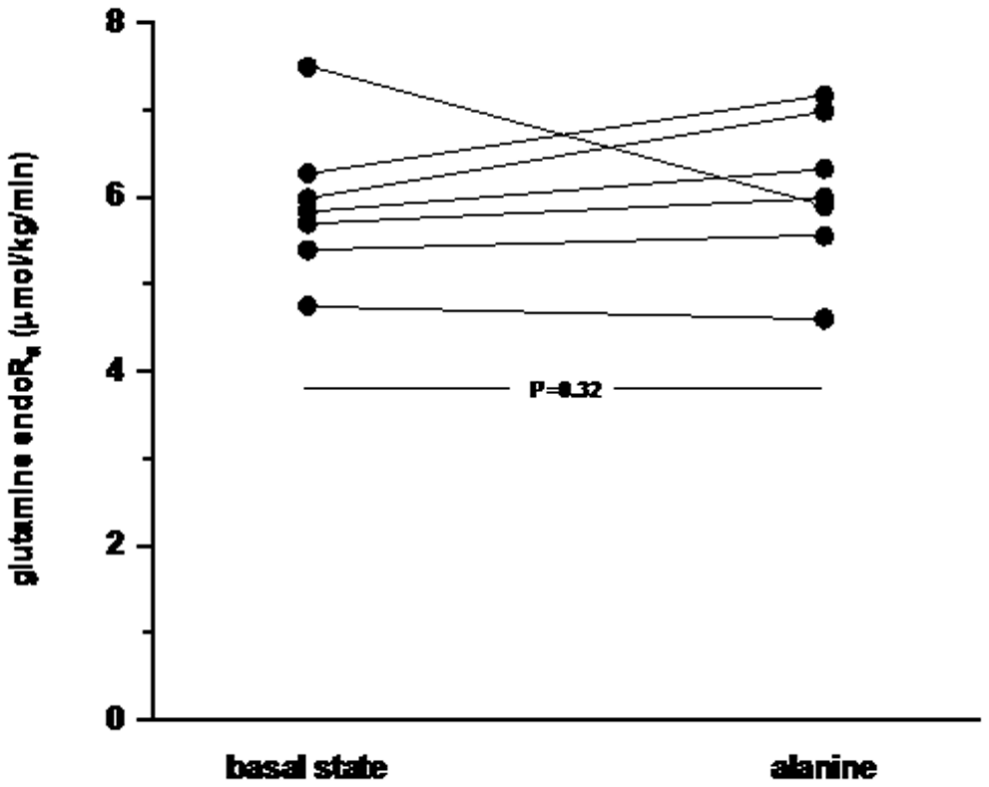
Endogenous glutamine R_a_ in healthy subjects (n = 7) studied before and during intravenous alanine supplementation.

During the 4-hour alanine infusion a steady state on plasma alanine concentration was reached after 2 hours (data not shown). Supplementation of alanine did not have any detectable effect on glutamine endoR_a_ (Figure 5 and Table S1). The glutamine endoR_a_ was 6.07±0.87 µmol/kg/min after the alanine infusion as compared to 5.90±0.85 µmol/kg/min before (P = 0.320).

## Discussion

Measuring glutamine kinetics in human has been difficult due to the large distribution volume of glutamine in skeletal muscle. Constant infusion of glutamine isotope, which is the conventional method for measuring endoR_a_, is associated with a relativly long measurement period even during optimal conditions [11]. In this study, we have investigated an alternative technique, a bolus injection method, followed by measuring the tracer enrichment decay curve. By sampling blood frequently after the bolus injection of glutamine tracer, the tracer enrichment in plasma declined over time in an ideal exponential curve, therefore estimation of glutamine endogenous production was possible for screening purposes by single pool modeling.

To determine the dose of [1-^13^C] glutamine, we tested 1.5, 3 or 6 mg/kg. All 3 doses of glutamine tracer resulted in an initial increase in the tracer enrichment, and then declined over time in an exponential curve. From these pilot data we decided to use a dose of 3 mg/kg in further studies since this gave us enrichments were detectable over 60 minutes that and only increased glutamine concentration slightly during the first 5 minutes. A 90 minutes sampling period was chose to assure that the tail of the decay-curve was not missed. When simulations were performed the sampling protocol chosen proved to be the most accurate taking both peak and tail of the decay-curve into account (Figure S1).

The glutamine endoR_a_ measured by 4 hours continuous infusion method is 5.2±0.2 µmol/kg/min (mean and SE) and 5.8±0.3 in young healthy subjects [1], [10], which is similar to that we found with the bolus injection, giving values ranging from 4.5 to 7.5 µmol/kg/min in the postabsorptive state. Also whether the continuous infusion of tracer was shorter or longer did not seem to have any crucial effect; endoR_a_ measured with a continuous infusion time of 8 hours versus 3 hours were 4.7±0.4 µmol/kg/min (mean and SE) and 5.7±0.4 µmol/kg/hour (mean and SE), respectively [13], [14] Furthermore, in a study measuring the glutamine endoR_a_ with the continuous infusion in 3 subjects and with the bolus injection in 3 subjects, no differences between the 2 approaches were observed [15]. It should be emphasized, however, that both techniques require an over all metabolic steady state to give reliable information over glutamine kinetics.

Glutamine endoR_a_ estimated by single pool modeling may be overestimated, due to the time needed for the bolus injection and the time point of the first blood sampling. It is most likely impossible to sample at the exact moment when the tracer enrichment has reached a maximum level in plasma, therefore the AUC of tracer enrichment is always smaller than can be expected. However, this overestimation is calculated to be low.

On the other hand, single pool model had a small CV when endoR_a_ was measured twice within the same individual. The GC-MS analytical CV was 4.1%, thus the CV of glutamine endoR_a_ calculated by single pool model, which was 5.5%, was mostly due to analytical variation. The reproducibility of glutamine endoR_a_ by single pool model was confirmed by performing 2 studies per subject. There was no difference between the 2 measurements performed 3 to 12 days apart.

Overall we could not find any difference of glutamine endoR_a_ between the measurements made with the continuous infusion and the bolus tracer infusion. Although frequent blood sampling is necessary, the time of study is much shorter than with the continuous infusion method, which will be an advantage in clinical studies. As discussed by Darmaun et al. [1] for the continuous infusion method the results most likely represent the transport of glutamine through the plasma pool rather than production rates due to the non-steady state in all pools. Since the bolus tracer infusion gives very similar results, this approach also seems to mostly represent the transport of glutamine through the plasma pool. A head-to-head comparison between the two techniques is associated with some problems; the times of measurement are different and different isotopes have to be used. Therefore we chose to compare numerical values and to validate by repeated measurements.

Our final goal is to measure glutamine endoR_a_ in ICU patients to understand the derangements of glutamine during critical illness better. In our institution, ICU patients are treated with alanyl-glutamine supplementation. Therefore we assumed that patient studies in the future will be performed under alanyl-glutamine supplementation and in the fed state. Thus the effects of alanyl-glutamine and parenteral feeding on glutamine kinetics were also examined in healthy subjects. With alanyl-glutamine supplementation, glutamine endoR_a_ did not change. However, when both TPN and alanyl-glutamine were given, the endoR_a_ of glutamine was significantly higher than in the control subjects in the postabsorptive state. In both groups that received the extra glutamine, the plasma glutamine levels were significantly higher than in the controls. These higher levels however did not feedback to the glutamine endoR_a_ by decreasing it, indicating that the production of glutamine is not controlled by the circulating glutamine levels. We have previously also observed this in critically ill patients given extra glutamine supplementation, at the same level as in the present study, with no effect on the amount of glutamine released from skeletal muscle [16], [17]. On the contrary, in the group receiving both alanyl-glutamine and parenteral nutrition, the endoR_a_ of glutamine was significantly increased, due to an increased release of glutamine into the plasma pool. This increase indicates an increased production of glutamine. Most likely this is the result of the increased delivery of precursors for glutamine synthesis in the form of other amino acids; in particular the branched chain amino acids. Despite the increased endoR_a_ of glutamine in this group, the plasma levels were not higher than in the group receiving the alanyl-glutamine only, which suggests that the flux of this extra glutamine through the plasma pool is increased and that the extra glutamine is utilized in other tissues than skeletal muscle.

Specifically we investigated whether alanine alone, which was given as part of the dipeptide in the earlier experiment, was a significant precursor for de novo glutamine synthesis, ie if an increased abundance of alanine in plasma when the ala-gln dipeptide was given would give an increase in glutamine endoR_a_. However, the result showed no difference in glutamine endoR_a_ in relation to a high dose of alanine given over 4 hours. We therefore conclude that the increase seen in glutamine endoR_a_ following dipeptide together with parenteral nutrition was not attributable to the alanine part of the dipeptide. The amino acid supplied by the parental nutrition is more likely to be the cause of the increased endoR_a_ of glutamine.

In conclusion, we validated a tracer bolus injection method to measure glutamine endoR_a_ in humans with good reproducibility and small variation. In addition, the method was valid during parenteral supplementation. We intend to apply this method to critically ill patients with and without extra glutamine and nutrition to establish potential derangements in their glutamine metabolism.

## Supporting Information

Figure S1 Endogenpous glutamine R_a_ calculated from simulation of data from subjects in control-group of sub-study “Alanyl-glutamine supplementation” (n = 5), testing different sampling protocols. Variations of sampling frequency during the peak as well as the length of sampling during the tail were considered. P-values for statistical differences from the default sampling protocol, No 1, are indicated.
(TIF)

Table S1 Glutamine kinetics data from all individual subject in all the sub-studies included in the publication.
(PDF)

## References

[1] Darmaun D, Matthews DE, Bier DM (1986) Glutamine and glutamate kinetics in humans. Am J Physiol 251: E117–126. 2873746 10.1152/ajpendo.1986.251.1.E117

[2] van Hall G, van der Vusse GJ, Soderlund K, Wagenmakers AJ (1995) Deamination of amino acids as a source for ammonia production in human skeletal muscle during prolonged exercise. J Physiol 489 (Pt 1): 251–261. 10.1113/jphysiol.1995.sp021047 PMC1156809 8583409

[3] van der Hulst RR, van Kreel BK, von Meyenfeldt MF, Brummer RJ, Arends JW, et al (1993) Glutamine and the preservation of gut integrity. Lancet 341: 1363–1365. 8098788 10.1016/0140-6736(93)90939-e

[4] Newsholme EA, Crabtree B, Ardawi MS (1985) Glutamine metabolism in lymphocytes: its biochemical, physiological and clinical importance. Q J Exp Physiol 70: 473–489. 3909197 10.1113/expphysiol.1985.sp002935

[5] Singleton KD, Wischmeyer PE (2007) Glutamine's protection against sepsis and lung injury is dependent on heat shock protein 70 expression. Am J Physiol Regul Integr Comp Physiol 292: R1839–1845. 17234954 10.1152/ajpregu.00755.2006

[6] Oudemans-van Straaten HM, Bosman RJ, Treskes M, van der Spoel H J, Zandstra DF (2001) Plasma glutamine depletion and patient outcome in acute ICU admissions. Intensive Care Med 27: 84–90. 11280678 10.1007/s001340000703

[7] Rodas PC, Rooyackers O, Hebert C, Norberg A, Wernerman J (2012) Glutamine and glutathione at ICU admission in relation to outcome. Clin Sci (Lond) 122: 591–597. 22248298 10.1042/CS20110520 PMC3294430

[8] Wernerman J, Kirketeig T, Andersson B, Berthelson H, Ersson A, et al (2011) Scandinavian glutamine trial: a pragmatic multi-centre randomised clinical trial of intensive care unit patients. Acta Anaesthesiol Scand 55: 812–818. 21658010 10.1111/j.1399-6576.2011.02453.x

[9] Heyland D, Muscedere J, Wischmeyer PE, Cook D, Jones G, et al (2013) A randomized trial of glutamine and antioxidants in critically ill patients. N Engl J Med 368: 1489–1497. 23594003 10.1056/NEJMoa1212722

[10] Jackson NC, Carroll PV, Russell-Jones DL, Sonksen PH, Treacher DF, et al (1999) The metabolic consequences of critical illness: acute effects on glutamine and protein metabolism. Am J Physiol 276: E163–170. 9886963 10.1152/ajpendo.1999.276.1.E163

[11] Van Acker BA, Hulsewe KW, Wagenmakers AJ, Deutz NE, Van Kreel BK, et al (1998) Absence of glutamine isotopic steady state: implications for the assessment of whole-body glutamine production rate. Clin Sci (Lond) 95: 339–346. 9730854

[12] Chaves Das Neves HJ, Vasconcelos AM (1987) Capillary gas chromatography of amino acids, including asparagine and glutamine: sensitive gas chromatographic-mass spectrometric and selected ion monitoring gas chromatographic-mass spectrometric detection of the N,O(S)-tert.-butyldimethylsilyl derivatives. J Chromatogr 392: 249–258. 3597576 10.1016/s0021-9673(01)94270-0

[13] Kreider ME, Stumvoll M, Meyer C, Overkamp D, Welle S, et al (1997) Steady-state and non-steady-state measurements of plasma glutamine turnover in humans. Am J Physiol 272: E621–627. 9142883 10.1152/ajpendo.1997.272.4.E621

[14] Matthews DE, Campbell RG (1992) The effect of dietary protein intake on glutamine and glutamate nitrogen metabolism in humans. Am J Clin Nutr 55: 963–970. 1570805 10.1093/ajcn/55.5.963

[15] Williams BD, Chinkes DL, Wolfe RR (1998) Alanine and glutamine kinetics at rest and during exercise in humans. Med Sci Sports Exerc 30: 1053–1058. 9662672 10.1097/00005768-199807000-00005

[16] Berg A, Bellander BM, Wanecek M, Norberg A, Ungerstedt U, et al (2008) The pattern of amino acid exchange across the brain is unaffected by intravenous glutamine supplementation in head trauma patients. Clin Nutr 27: 816–821. 18649974 10.1016/j.clnu.2008.06.006

[17] Berg A, Norberg A, Martling CR, Gamrin L, Rooyackers O, et al (2007) Glutamine kinetics during intravenous glutamine supplementation in ICU patients on continuous renal replacement therapy. Intensive Care Med 33: 660–666. 17318498 10.1007/s00134-007-0547-9

