# Supplementary material for: A Tracer Bolus Method for Investigating Glutamine Kinetics in Humans
Source: PLoS One. 2014 May 8;9(5):e96601. doi: 10.1371/journal.pone.0096601 (PMC4014541; doi:10.1371/journal.pone.0096601)
Supplement: Table S1 — Glutamine kinetics data from all individual subject in all the sub-studies included in the publication. (PDF) [file pone.0096601.s002.pdf]

Supplementary table 1 to: A tracer bolus method for investigating glutamine kinetics in humans. By M Mori, M Smedberg, M Klaude, ITjäder, Å Norberg, O Roc
Individual glutamine kinetics data from all studies in publication

**Study:**	**Interventions**			**Kinetics data**			
**subject**	**TPN**	**Ala-Gln**	**Ala**	**Dose**	**AUC**	**Peak**	**endogenous R_a**
	+/-	+/-	+/-	µmol/kg ^{13}C-glutamine	APE	APE	µmol/kg/min glutamine
**Variation**[1]							
**1.1**	-	-	-	20.3	366	49.2	5.0
**1.2**	-	-	-	20.3	330	53.9	5.5
**2.1**	-	-	-	20.5	333	60.7	5.5
**2.2**	-	-	-	20.7	374	66.8	5.0
**3.1**	-	-	-	20.1	347	53.6	5.2
**3.2**	-	-	-	20.5	358	49.4	5.2
**4.1**	-	-	-	20.4	354	50.2	5.2
**4.2**	-	-	-	20.0	312	57.7	5.8
**Supplementation**							
**CON**							
**1**	-	-	-	19.3	269	51.0	6.5
**2**	-	-	-	20.5	325	70.0	5.7
**3**	-	-	-	19.5	377	51.0	4.7
**4**	-	-	-	20.8	270	53.7	6.9
**5**	-	-	-	21.0	289	48.2	6.6
**GLN**							
**1**	-	+	-	21.0	211	48.4	7.0
**2**	-	+	-	20.3	177	46.2	8.4
**3**	-	+	-	20.6	233	36.1	6.0
**4**	-	+	-	16.9	195	38.6	5.9
**5**	-	+	-	20.8	205	39.2	7.2
**GLN+TPN**							
**1**	+	+	-	20.0	171	45.9	8.7
**2**	+	+	-	20.4	226	64.6	6.2
**3**	+	+	-	20.5	206	52.3	7.0
**4**	+	+	-	20.5	191	23.0	7.7
**5**	+	+	-	17.8	180	36.5	7.0
**6**	+	+	-	21.3	190	35.6	8.2

Alanine[1]

**1.1**	-	-	+	20.5	311	47.0	5.9
**1.2**	-	-	+	21.6	279	39.8	7.0
**2.1**	-	-	+	20.4	341	64.8	5.4
**2.2**	-	-	+	20.0	324	53.0	5.6
**3.1**	-	-	+	19.6	235	48.2	7.5
**3.2**	-	-	+	18.1	276	44.6	5.9
**4.1**	-	-	+	20.3	383	60.6	4.8
**4.2**	-	-	+	20.2	396	54.4	4.6
**5.1**	-	-	+	20.1	318	49.8	5.7
**5.2**	-	-	+	20.0	300	46.2	6.0
**6.1**	-	-	+	19.2	276	46.1	6.3
**6.2**	-	-	+	19.8	249	39.4	7.2
**7.1**	-	-	+	20.0	309	45.4	5.8
**7.2**	-	-	+	19.7	280	41.8	6.3

Notes:
1) two studies per subject

›yackers, J Wernerman
